# Study of vertebral fracture and Scanographic Bone Attenuation Coefficient in rheumatoid arthritis and ankylosing spondylitis *vs*. controls

**DOI:** 10.1038/s41598-019-49712-x

**Published:** 2019-09-16

**Authors:** Marine Fauny, Eliane Albuisson, Elodie Bauer, Julia Perrier-Cornet, Isabelle Chary-Valckenaere, Damien Loeuille

**Affiliations:** 1 Department of Rheumatology, University Hospital, Nancy, France; 2 Pôle S2R, PARC, University Hospital, Vandoeuvre lès Nancy, France; 3 University of Lorraine, Faculty of Medicine, InSciDens, Vandoeuvre lès Nancy, France; 4 CNRS, Institute Elie Cartan de Lorraine, UMR 7502, Vandoeuvre-lès-Nancy, F-54506 France

**Keywords:** Rheumatoid arthritis, Ankylosing spondylitis

## Abstract

The objective of this study is to identify the prevalence of vertebral fractures (VFs) and to measure the scanographic bone attenuation coefficient of the first lumbar vertebra (SBAC-L1) based CT-scan, a biomarker of bone fragility in patients with rheumatoid arthritis (RA) and ankylosing spondylitis (AS) and in a control group. This monocentric and retrospective study included patients with RA and AS, based on ACR/EULAR or New-York criteria, respectively. A control group was constituted. All of the patients received a CT-scan. VFs were determined via CT-scans according to the Genant classification, and the SBAC-L1 was measured in Hounsfield units (HU). SBAC-L1 ≤145 HU (fracture threshold) defined patients at risk of VFs. 244 patients were included (105 RA, 83 AS, 56 controls). Of the 4.365 vertebrae studied, 66 osteoporotic VFs were found in 36 patients: 18 (17.1%) RA, 13 (15.7%) AS and 5 (8.9%) controls. The mean SBAC-L1 was 142.2 (±48.4) HU for RA, 142.8 (±48.2) for AS, both of which were significantly lower than that of the control group (161.8 (±42.7) HU). Of the 36 patients with VFs and rheumatism, 28% had a T-score ≤−2.5 SD and 71.4% a SBAC-L1 ≤145 HU. A T-score ≤−2.5 SD and a SBAC-L1 ≤145 HU were associated with VF (OR = 3.07 (CI 95%: 1.07; 8.81), and 2.31 (CI 95%: 1.06; 5.06)), respectively. The SBAC-L1 was significantly lower in the RA and AS groups than in the control group. Furthermore, SBAC-L1 ≤145 HU was associated with a higher risk of VFs, with an odds ratio similar to that of a DXA.

## Introduction

Osteoporosis is a common disease whose prognosis can be seriously impacted by the development of fractures that lead to functional limitations and may even have life-threatening sequelae^1^. This disease is often under-screened, especially in at-risk populations that require multidisciplinary care such as patients with rheumatoid arthritis (RA) and ankylosing spondylitis (AS). The risk of bone fracture is well documented for patients with RA^2^. However, when there are other associated comorbidities (e.g., cardiovascular, infectious, cancerous…), bone screening is often relegated to the background due to the emphasis that is placed on treating the active disease^2,3^. AS has a predilection for younger males, and the risk of osteoporosis associated with this disease is widely accepted^4–8^. Factors that contribute to bone fragility in these diseases include systemic inflammation, sedentariness, menopause, iatrogenic causes, especially corticosteroids in the case of RA and to the presence of subclinical intestinal inflammatory involvement for patients with AS^9–15^.

Thoracic or thoraco-abdomino-pelvic (TAP) computed tomography scan (CT-scan) can be used to evaluate VFs from C7 to L1 with a thoracic CT-scan and from C7 or L1 to S1 with a thoraco-abdomino-pelvic or an abdomino-pelvic CT-scan. The first lumbar vertebra is available on a thoracic CT scan and is easily identified as the first vertebra without rib-bearing. Moreover, this vertebra is evaluated on DXA with the other lumbar vertebrae, excepted the fifth (L1 to L4). To finish, a recent study^16^ reported that a scanographic bone attenuation coefficient of the first lumbar vertebra (SBAC-L1) ≤145 HU (Hounsfield Units) was more sensitive than a T-score ≤−2.5 SD for identifying vertebral fracture risk. The SBAC-L1 identified 96.6% of the patients with a higher risk of VFs, whereas the DXA (with a T-score ≤−2.5 Standard Deviation (SD)) identified only 39%. CT-scans are often performed in patients with RA or AS and are often employed in the general population to evaluate complications or intercurrent events (infectious, cancerous…) that may or may not be associated with immune-suppressive treatment.

The aim of this this work was to determine, on CT-scan, the prevalence of VFs, to measure the SBAC-L1 and to establish the prevalence of patients with RA and AS who are under the fracture threshold of 145 HU. Comparisons between the two populations were made with a RA-matched control group. Finally, we have examined the association between a low SBAC-L1 and the presence of one or more VFs in these different populations.

## Methods

### Population

This descriptive and retrospective study included 3 populations:

- **Rheumatoid arthritis (RA):**
  Patients with RA, based on the ACR/EULAR (American College of Rheumatology/European League Against Rheumatism) criteria, followed at the University Hospital of Nancy between January 2010 and December 2014 were included if they had a CT-scan and DXA (with a maximum gap of 2 years between the 2 exams) during their follow-up.
- **Ankylosing spondylitis (AS):**
  Patients with AS based on the New-Work criteria with radiographic evidence of sacroiliitis (with or without structural involvement of the spine), followed at the University Hospital of Nancy between September 2009 and 2017 were included if they had a CT-scan during their follow-up.
- A control group was composed of individuals with demographic characteristics similar to the RA population (RA is the only rheumatism recognized as a full osteoporotic risk factor^2^) and no past medical history of inflammatory disease or osteoporosis. Patients were included if they had a CT-scan during their follow-up. These patients received their care at the University Hospital of Nancy (rheumatology, internal medicine, infectious diseases, dermatology and geriatric departments) or at the rehabilitation centre of Nancy between May and October 2017.

Demographic characteristics (age, gender, smoking…), clinical informations (disease duration…), biological data (C-reactive protein (CRP)) and informations about therapeutic treatments (corticosteroids) were collected from the complete medical record.

For the evaluation of osteoporosis, clinical risk factors (gender, age, biological inflammation, smoking and corticosteroids), spine DXA data and therapeutic data were collected for all patients with RA and AS. For the control group, only the clinical risk factors were collected.

**Figure 1:**
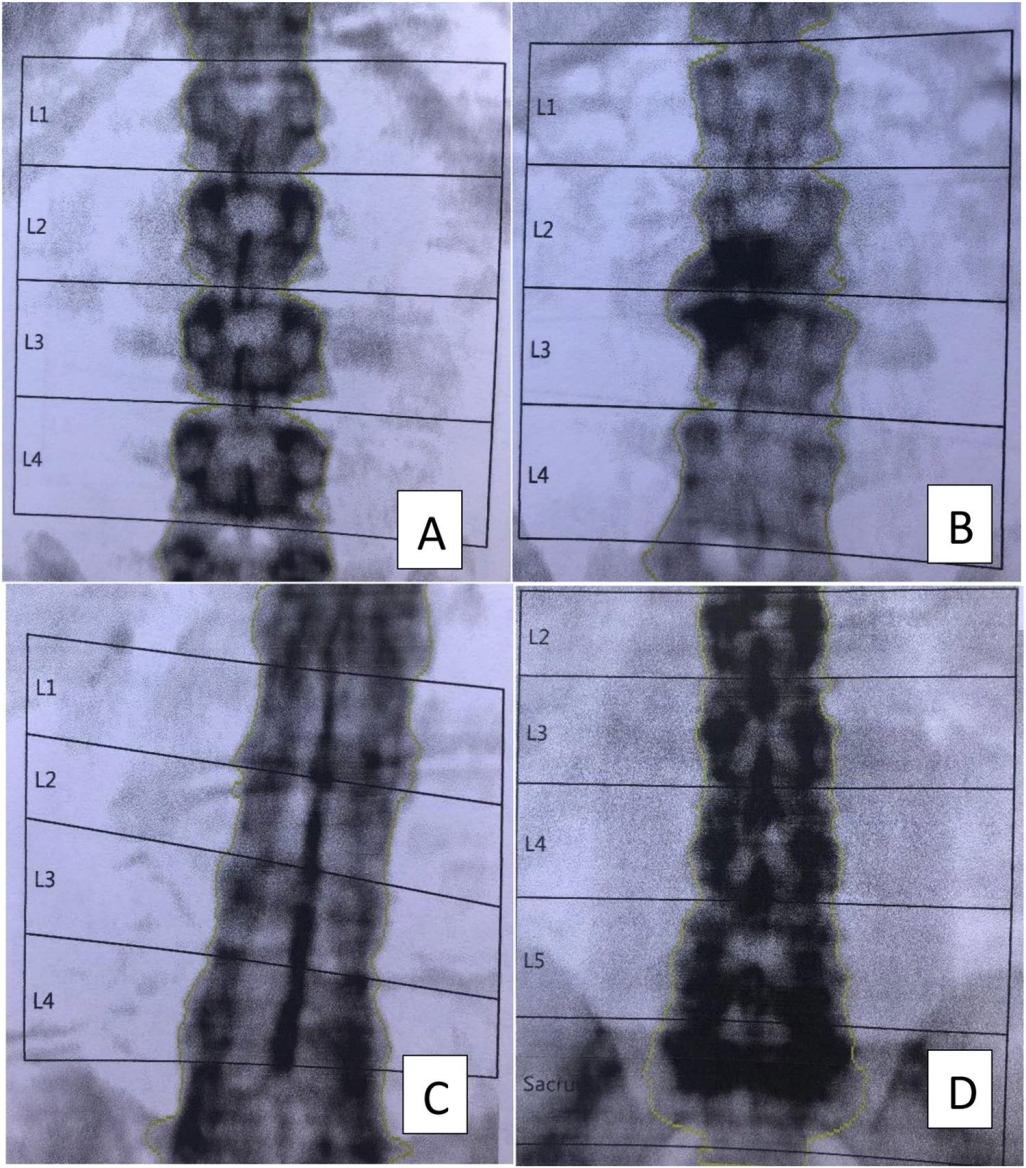
Spine DXA examples. (**A**) Spine DXA from patients without lumbar spine involvement. (**B**) Spine DXA from patient with osteophytes in L2 and L3 responsible for increase of spine mineral density on spine DXA. (**C**,**D**) DXA from patients with ankylosed spine, source of misinterpretation for DXA spine results.

Osteoporosis is classically defined as a T-score ≤−2.5 SD (standard deviation). DXA values included lumbar vertebra bone mineral density from L1 to L4, excluding vertebra with fracture. Presence of vascular calcic deposits, degenerative vertebra damages such as osteophytes, have taken into account to interpret the results and we excluded abnormal vertebra (Fig. 1).

### Morphological and densitometric scanographic bone evaluation (vertebral fracture and scanographic bone attenuation coefficient of L1: SBAC-L1)

All the CT-scans were performed at the University Hospital of Nancy and were read using OSIRIX software (v6.5.1–64 bits).

The SBAC-L1 study was conducted on L1 axial sections through the pedicles on the bone window. The largest elliptical Region of Interest (ROI) was drawn in the trabecular bone and provided the average bone mineral density (in HU). If there was a VF in the L1 vertebra, the measurement was performed on the adjacent vertebrae (CT-scan performed similarly from T12 to L5)^16^. This evaluation was performed by a single lector (MF) because we had previously demonstrated the excellent reliability on this measure in an earlier study (for intra and inter-reader, kappa >0.9)^17^.

The VFs were analysed manually on the sagittal reconstruction in bone windows and based on an adaptation of the Genant classification, which is usually used for spine radiographs^18^. The grade was determined based on the most severe lesion observed on one of the sagittal sections. We studied the vertebrae from C7 to L1 with the thoracic CT-scan and from C7 or L1 to S1 with the thoraco-abdomino-pelvic or abdomino-pelvic CT-scan. The lumbar CT-scan was used to study the region from L1 to L5. Two independent rheumatologists who were blinded to the clinical data interpreted the results. Any disagreement was resolved with the input a third rheumatologist. We then studied only the significant VFs (grades 2 and 3).

This threshold of 145 HU was used in our three populations to maintain an acceptable balance between sensitivity and specificity^16^.

We examined the association between a low SBAC-L1 or a diagnosis of osteoporosis on DXA (T-score ≤−2.5 SD) and the presence of VFs in these groups.

### Ethics approval

All of the data used were obtained from the medical records. No examinations were performed for patients to meet the inclusion criteria. This study is registered to the Information Technology and Freedoms Commission for the University Hospital of Nancy (File Number: R2018–12) and was designed in accordance with the general ethical principles outlined in the Declaration of Helsinki. The protocol of this study was approved by the Information Technology and Freedoms Commission for the University Hospital of Nancy. All patients gave their consent for the use their medical data during the time period they received medical care at the University Hospital.

### Statistical analysis

Both descriptive and comparative analyses were conducted by accounting for the nature and distribution of the variables. Qualitative variables are described with frequencies and percentages; quantitative variables are reported as the mean ± SD (standard deviation) or as the median and interquartile range (IQR). The Kolmogorov-Smirnov test showed that among the continuous demographic and clinical variables, only age and spinal bone mineral density followed a normal distribution. For the comparisons, ANOVA was used to analyse age, Student’s t-test was used for the spinal bone mineral density, the Mann-Whitney U test for the disease duration and the Kruskal-Wallis test was used for BMI and SBAC-L1. For qualitative variables, the chi-square test with Fisher’s exact test, if necessary, was used. Only significant results are presented with odds ratio (OR) and their 95% confidence interval (CI 95%), calculated through univariate logistic regression. To analyse the intra-reader and the inter-reader reliability, we used the Cohen’s kappa. The risk α was established as 0.05, except for the inter-reader reliability study, where it was established at 0.01 given the repetition of the tests. IBM SPSS Statistics V22 was the software used for the data analysis.

## Results

### Population

**Figure 2:**
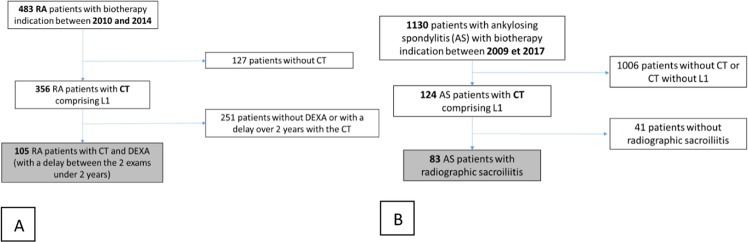
Flow chart for the group RA (**A**) and AS (**B**) AS: ankylosing spondylitis, RA: Rheumatoid Arthritis; CT: Computed Tomography.

A total of 105 patients with rheumatoid arthritis (RA), 83 patients with ankylosing spondylitis (AS) and 56 controls were included (Fig. 2A,B).

### Demographic data and osteoporotic risk assessment

The AS group had a lower mean age (56.5 years) and an inverse sex ratio (86.7% men, 13.3% women) compared with the other groups. Only 29 patients (34%) in the AS group had a spine DXA. All of the patients with RA had at least two clinical risk factors for osteoporosis, while 32.5% of the patients with AS had at least two risk factors, and 61 (73.5%) had at least one risk factor. Only 3.6% of the control group had an osteoporosis risk factor (Table 1).

**Table 1:** Demographic data from the different populations.

	RA	AS	Control	P
	N = 105	N = 83	N = 56	
**Demographic characteristics**				
Age_(mean(SD))_	61.1 (9.5)	56.5 (10.76)*	65.2 (12.6)	**0.0001**
Women_(N(%))_	82 (78.1)	11 (13.3)*	49 (87.5)	**0.0001**
Smoking_(N(%))_	51 (48.6)	37 (44.6)	5 (8.9)**	**0.0001**
Disease duration_(median(IQR))_	12.0 (7: 20.5)	21 (9.5: 30)	/	**0.002**
Biological inflammation_(N(%))_	70 (66.7)	36 (43.4)	/	**0.004**
Corticosteroid therapy_(N(%))_	86 (81.9)	11 (13.25)	/	**0.0001**
BMI_(median(IQR))_	26.1 (22.6: 32.4)	25.4 (23.3: 30.9)	27.4 (23.1: 37.6)	0.384
**Data on osteoporosis**				
Number of spine DXA_(N(%))_	104 (99)	29 (34.9)	/	**0.0001**
Osteoporosis on DXA (N (%))	28 (26.7)	5 (6.02)	/	0.22
Mean BMD on spine_(mean(SD))_	1.07 (0.2)	1.16 (0.24)	/	**0.036**
Clinical osteoporosis risk factor ≥2_(N(%))_	105 (100)^‡^	27 (32.5)	2 (3.6)	**0.0001**
Calcium and/or Vitamin D_(N(%))_	42 (40)^‡^	18 (21.68)	4 (7.1)	**0.0001**
Specific treatment_(N(%))_	37 (35.2)^†^	13 (15.66)	0 (0)	**0.002**

RA: Rheumatoid Arthritis, AS: Ankylosing spondylitis, DXA: Dual Energy X-ray Absorptiometry, BMI: Body Mass Index.

^‡^Significant difference between RA and the other groups, p < 0.0001.

^†^Significant difference between RA and the other groups, p = 0.002.

*Significant difference between AS and the other groups, p = 0.0001.

**Significant difference between Control group and the other groups, p = 0.0001.

Compared with the other groups, antiosteoporotic treatments were more often prescribed for patients with RA such as vitamin D and/or calcium (28.6%) or a specific treatment for osteoporosis (30.4%) (p = 0.0001 and 0.002, respectively).

### Vertebral fracture (VF) prevalence in the different groups

Two-hundred and forty-four CT-scans were evaluated: 10 thoracic, 217 thoraco-abdomino-pelvic and 17 abdomino-pelvic.

**Figure 3:**
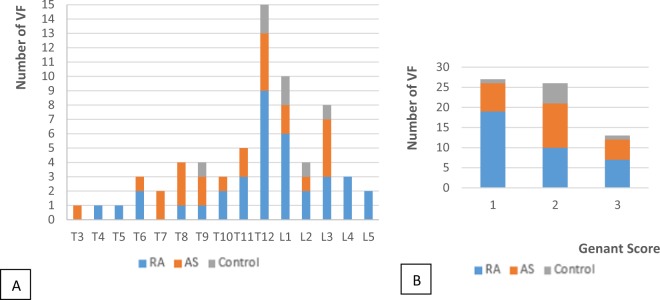
VFs grade (Genant classification) (**A**) and location (**B**) according to the different groups. AS: ankylosing spondylitis, RA: Rheumatoid Arthritis. T: Thoracic vertebra, L: Lumbar vertebra.

A total of 4,365 vertebrae were evaluated with 66 VFs (all grades). The inter-reader reproducibility was excellent in all of the populations for the diagnosis of VFs on CT-scan (Kappa for AS: 0.88, for control: 0.96 and for RA: 0.92). In the RA group, we identified 32 VFs, 23 in the AS group and 7 in the control group, with a prevalence of 17.1%, 15.7% and 8.9% for the RA, AS and control groups, respectively. These fractures involved both the thoracic and lumbar vertebrae though they were predominantly at the thoraco-lumbar junction (Fig. 3A and Table 2). Grade 1 fractures predominated in the RA group (70% of the grade 1). Grades 2 and 3 represented 59.1% of the total VFs (Fig. 3B).

**Table 2:** Results of the osteoporosis risk assessment according to the different populations.

	RA	AS	Control	P
	N = 105	N = 83	N = 56	
Mean SBAC-L1_(median(IQR))_	135.2 (109.3: 175.6)	140.1 (109.5: 166.1)	156.7^‡^ (130.7: 183.3)	**0.016**
SBAC-L1 ≤145 HU_(N(%))_	64 (61)	46 (55.4)	21 (37.5)^†^	**0.007**
Number of VFs_(N)_	36	23	7	0.36
Patients with VFs_(N(%))_	18 (17.1)	13 (15.7)	5 (8.9)	0.36
Patients with VFs (Genant ≥2)_(N(%))_	9 (8.6)	11 (13.25)	4 (7.14)	0.42
Osteoporosis on DXA (spine)_(N(%))_	28 (26.7)	5 (6.02)	/	0.22
BMD on spine_(mean(SD))_	1.07 (0.2)	1.16 (0.24)	/	**0.036**

RA: Rheumatoid Arthritis, AS: ankylosing spondylitis.

SBAC-L1: Scanographic Bone Attenuation Coefficient of the first lumbar vertebra, HU: Hounsfield Unit, VF: Vertebral fracture, DXA: Dual Energy X-ray Absorptiometry, BMD: Bone Mineral Density.

^‡^Significant difference between the control group and the other groups, p = 0.016.

^†^Significant difference between the control group and the other groups, p = 0.007.

### Scanographic bone attenuation coefficient of L1 (SBAC-L1) evaluation

Four measures of the SBAC-L1 were not available in the RA group for technical reasons, and one was unavailable in the AS group due to a deformity of the spine that prevented the placement of a ROI in the trabecular bone.

The mean SBAC-L1 was 142.2, 142.8 and 161.3 HU in the RA, AS and control groups, respectively. Sixty-one percent of patients with RA and 55.4% of patients with AS had a SBAC-L1 under the fracture threshold compared with 37.5% of controls (Table 2).

The SBAC-L1 of patients with a chronic inflammatory disease (RA and AS) was significantly lower than that of controls (p = 0.016), and more patients with RA and AS had a SBAC-L1 under the fracture threshold of 145 HU (p = 0.007).

In total, 36 patients had at least one VF: 18 (17.1%) in the RA group, 13 (15.7%) in the AS group and 5 (8.9%) in the control group. There was no significant difference between the groups. When considering only grades 2 and 3, 24 patients had VFs: 9 (8.6%) in the RA group, 11 (13.2%) in the AS group and 4 (7.4%) in the control group.

### Vertebral fractures (VFs) and relationship with T-score and scanographic bone attenuation coefficient (SBAC-L1)

#### VFs and SBAC-L1

**Figure 4:**
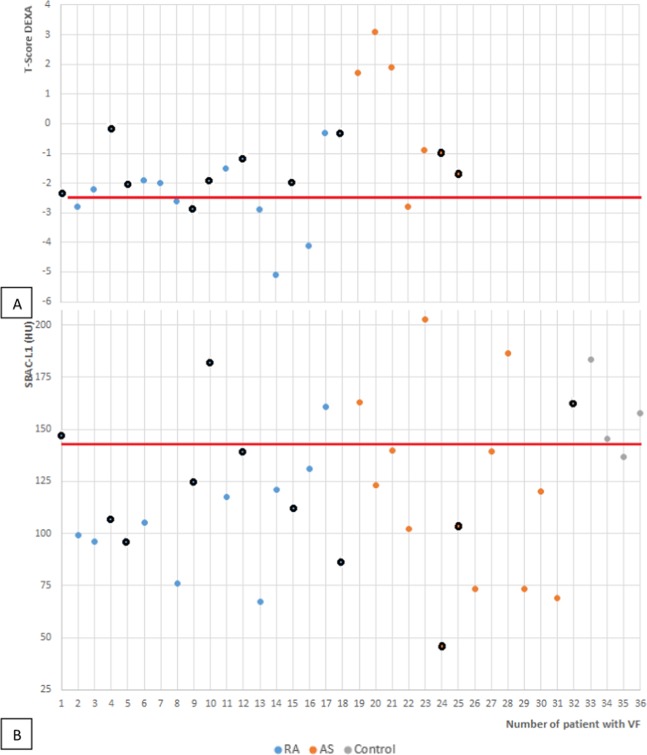
Spine T-score (**A**) and SBAC-L1 (**B**) distribution of the patients with VFs for the different groups. The black circle represents the grade 1 VFs. (**A**) The red line stands for the threshold of −2.5 SD, the definition of osteoporosis on DEXA. (**B**) The red line stands for the fracture threshold of 145 HU. Note that patient no. 7 did not have an available SBAC-L1. AS: Ankylosing spondylitis, RA: Rheumatoid Arthritis. VF: Vertebral Fracture, SBAC-L1: Scanographic Bone Attenuation Coefficient of the first lumbar vertebra, HU: Hounsfield Unit, DEXA: Dual Energy X-ray Absorptiometry.

The SBAC-L1 was available for 35/36 (97.2%) patients with VFs. 25 of these patients (71.4%) have aSBAC-L1 under the fracture threshold (≤145 HU) (Fig. 4B): 14 of 17 patients with VFs in the RA group, 10 of 13 patients in the AS group and 1 of 5 controls.

#### VFs and T-score for the spine

25/31 (80.6%) patients with inflammatory rheumatic disease had a spine DXA available. Seven patients with VFs (28%) had osteoporosis on DXA. Seven (53.8%) of the 13 AS patients with VF had a DXA; one had osteoporosis (T-score ≤−2.5 SD), and one had a low bone mass (1 >T-score >−2.5 SD). Of 18 patients with RA and a VF, 6 had osteoporosis on DXA, 9 had a low bone mass and 3 had normal bone mass (Fig. 4A).

A SBAC-L1 ≤145 HU and a T-score ≤−2.5 SD were associated with VF with an OR of 2.31 (CI 95%:1.06; 5.06) and 3.07 (CI 95%:1.07; 8.81), respectively.

## Discussion

This is the first study to evaluate the presence of VFs and bone fragility (SBAC-L1) using CT-scans performed during routine care, in populations at risk of osteoporosis (namely, patients with RA and AS) and in a control group. We showed that the prevalence of VFs was similar in patients with RA and AS and tended to be higher than RA-matched control patients, but results did not reach the statistical significance related to a lack of power. We also showed in these 2 populations at risk of VFs the SBAC-L1 was lower compared with controls, suggesting the presence of higher bone fragility. Furthermore, the CT-scan appeared to be as reliable as the spine DXA for screening patients with inflammatory rheumatic disease at risk of VFs. In fact, a SBAC-L1 ≤145 HU and a T-score ≤−2.5 SD were associated with VF with OR of 2.31 (CI 95%: 1.06; 5.06) and 3.07 (CI 95%: 1.07; 8.81), respectively. The CT-scan was obtained during the patients’ follow-up. At the University Hospital of Nancy, approximately 24% of patients with AS and 70% of patients with RA undergo a CT-scan before receiving a biologic treatment because of the presence of comorbidities (cardiovascular, cancerous, infectious), advanced age or treatment-related complications during their follow-up.

With respect to the specific populations, the AS group was composed of a greater number of younger males than were the other groups and included only patients with radiographic sacroiliitis. As a consequence, the heterogeneity in this population was reduced (major demographic and clinical differences between patients with or without radiographic sacroiliitis). In the RA group, menopausal females predominated, and the patients were treated more frequently with corticosteroids (81.9%)^2,3^. The demographic characteristics of the control group were subject to adjustment with RA group since RA is the only rheumatic inflammatory disease recognized as a full osteoporosis risk factor^2^. Thus, the populations were also not strictly comparable in terms of the risk factor for osteoporosis. Indeed, we considered the younger male population to have fewer clinical risk factors.

In our study, the prevalence of osteoporosis on DXA was 26.7% and 6.02% in the RA and AS groups, respectively. However, data was missing because only 34.9% of patients with AS had a DXA. In the literature, the prevalence of osteoporosis on DXA varies depending of the population. The prevalence varies between 24.5% and 41.1% for patients with RA^19–21^. In our study, the prevalence of osteoporosis on DXA among patients with AS of 6.02% is lower than that reported in previous studies, in which the prevalence varied between 13.4% and 50%, though this included both AS and non-radiographic spondyloarthritis^5,7,22–24^. In women over 45 years in the general population, from INSTANT cohort, the overall prevalence of diagnosed osteoporosis is reported to be 9.7%, but increased according to the age, from 1.6% for the 45–50 year old patients to 17.2% for patients aged 70–74 years. Over 75 years, the prevalence rate remained relatively stable and tended to slightly decrease after 85 years-old^25^. This prevalence also varies according to the sex: osteoporosis is 2 to 3 times higher in women than men. The prevalence of osteoporosis was 1.6% in men over 65 years for Ferrari^26^ and concern 4 to 6% of men over 50 years in another study^27^.

With respect to the scanographic evaluation, we reviewed 244 CT-scans (10 thoracic, 217 thoraco-abdomino-pelvic and 17 abdomino-pelvic). For 10 (4.1%) patients, the study was only performed on the thoracic vertebrae (including L1) and only on the lumbar segment for 17 (6.9%) patients. Thus, we may be underestimating the prevalence of VFs because, as described in the literature, VFs are often located in the thoraco-lumbar hinge or in the upper lumbar spine^28^.

We found a prevalence of VFs of 17.1%, 15.7% and 8.9% in the RA, AS and control groups, respectively. We have previously reported prevalence of VF and SBAC-L1 measurement in a population of RA patients followed by CY-scan^29^. But to our knowledge, no study had compared VF prevalence in RA, AS and RA-matched control patients. So, when we considered studies where the radiography was the criteria of judgment, the prevalence of VFs varies between 15 and 45% for RA^30–34^, between 0.86 to 31% for AS^5,22,24,35^ and 12%^36^ in the general population. The relative risk varies between 1.52 to 2.93 for RA^37–39^, and between 1.96 to 7.1 for AS^35,39^. The heterogeneity of these results is due to the different methods used to evaluate VFs across the studies (radiography, vertebral fracture assessment (VFA)) as well as the method of patient recruitment. A significant portion of VFs are asymptomatic and require complementary examinations to be identified. All of our results were in accordance with those in previous studies that used DXA to diagnose osteoporosis and radiographies or VFA to diagnose VFs.

We studied the prevalence of patients with a SBAC-L1 under the 145 HU fracture threshold, as proposed by Pickardt to achieve a compromise between sensitivity and specificity^16^. This measurement, such as that of DXA, is very reproducible (both intra- and in inter-reader). The bone fragility prevalence on CT-scan was 58.1%, 55.4% and 37.5% for RA, AS and the control groups, respectively, which is higher than that with DXA (26.7% and 6.0% for RA and AS, respectively). A SBAC-L1 ≤145 HU and a T-score ≤−2.5 SD were associated with VF with OR of 2.31 (CI 95%: 1.06; 5.06) and 3.07 (CI 95%: 1.07; 8.81), respectively.

Our assessment tools, VF evaluation and SBAC-L1 measures were very reproducible. The CT-scan allows a morphological study (VF) and a bone density evaluation (SBAC-L1) to be completed in a single exam. In our study, 71.4% of patients with VFs have a SBAC-L1 under the fracture threshold, whereas only 28% have osteoporosis on DXA (with a T-score ≤−2.5 SD). Though DXA is the gold standard for osteoporosis screening, it has some known limitations such as an overestimation of values in cases of degenerative or structural damage, vascular calcifications, and renal calculi. Furthermore, the assessment is only quantitative and not qualitative. The DXA includes an assessment of both cortical and trabecular bone, while the CT-scan allows for an evaluation of the trabecular bone alone, avoiding cortical spine damage. In the AS group, only one patient with VF was diagnosed with osteoporosis on the 7 AS patients with available DXA. Indeed, patients with AS may have syndesmophytes or ankylosis (ectopic bone production) spearhead an over-estimation of DXA and so causing an under-estimation of the osteoporosis risk. Bone fragility screened by the SBAC-L1 seems of interest in AS patients where cortical bone formation on vertebra corners may be a cause of false negative DXA results.

These results support the pathophysiological aetiology of bone loss, which increases in the presence of systemic inflammation^40^ such as observed in RA or AS. This decrease in bone density is multifactorial. While general factors, such as race, age, gender, BMI, smoking, alcohol consumption, menopause or family or personal history of fracture are all contributors, there are additional specific factors (in relation to the disease) such as corticosteroid treatment, sedentariness, disease activity or duration and structural damage that are also impactful. The pathophysiology underlying AS is more complex because bone resorption (with erosion such as Romanus’ osteitis) and bone formation (syndesmophyte, ankylosis…) occur simultaneously, even though these phenomena are often uncoupled. The bone loss is due to the interaction of inflammatory mediators (IL, cytokines…) with bone cells leading to the activation of the osteoclasts through the RANK/RANK-L system^41^. A loss of mobility of spine segments is also another parameter to consider.

The contribution of CT-scan and the development of low dose spine acquisitions offer new possibilities for evaluating patients with rheumatic disease and should lead to a better VFs risk evaluation and screening of bone fragility (apprehended by SBAC-L1). Indeed, the last recommendations for comorbidity screening in rheumatism disorders always underscore osteoporosis screening in the follow-up^8^.

In conclusion, the prevalence of VFs was similar in patients with RA and AS. We also showed in these 2 populations at risk of VFs the SBAC-L1 was lower compared with controls, suggesting the presence of higher bone fragility. Furthermore, the CT-scan appeared to be as reliable as the spine DXA for screening patients with inflammatory rheumatic disease at risk of VFs. In fact, a SBAC-L1 ≤145 HU and a T-score ≤−2.5 SD were associated with VF with OR around 2.
